# Artificial Intelligence Use in Medical Education: Best Practices and Future Directions

**DOI:** 10.1007/s11934-025-01277-1

**Published:** 2025-05-29

**Authors:** Rasheed A. M. Thompson, Yash B. Shah, Francisco Aguirre, Courtney Stewart, Costas D. Lallas, Mihir S. Shah

**Affiliations:** Department of Urology, Sidney Kimmel Medical College, Thomas Jefferson University, Philadelphia, PA United States

**Keywords:** Artificial Intelligence, Medical Education

## Abstract

**Purposeof Review:**

This review examines the various ways artificial intelligence (AI) has been utilized in medical education (MedEd)and presents ideas that will ethically and effectively leverage AI in enhancing the learning experience of medical trainees.

**Recent Findings:**

AI has improved accessibility to learning material in a manner that engages the wider population. It has utility as a reference tool and can assist academic writing by generating outlines, summaries and identifying relevant reference articles.

**Summary:**

As AI is increasingly integrated into MedEd and practice, its regulation should become a priority to prevent drawbacks to the education of trainees. By involving physicians in AI design and development, we can best preserve the integrity, quality, and clinical relevance of AI-generated content. In adopting the best practices for AI use, we can maximize its benefits while preserving the ethical standards of MedEd with the goal of improving learning outcomes.

## Introduction

The emergence of artificial intelligence (AI) has had a notable impact on everyday life. Particularly in medicine, AI can now diagnose cancer through radiographic imaging, personalize medical care, and even answer patient questions [1–6]. Additionally, AI can reduce the documentation burden and minimize human error by closely monitoring clinical decisions and automating portions of medical administration [7–9]. Recently, we have seen the emergence of AI tools supporting medical education (MedEd). While the benefits of AI are promising, its integration in MedEd creates great debate regarding the best practices for use, with concerns of interference with critical thinking, creativity, and research integrity.

Since its inception, AI has revealed itself to be widely adaptable, yielding great benefits in various scenarios. Existing literature on AI applications for MedEd has focused on personalized feedback, particularly in surgical settings and medical training as a whole [10–12]. Feedback is crucial for trainees'continued learning and for preserving the delivery of quality care. [10, 11] AI has enhanced this process by increasing the efficiency with which feedback is delivered to trainees. Other useful applications include tutoring for standardized exams and surgical skills development.

As AI becomes embedded across various sectors in our society, we must proactively study the benefits and risks associated with its use. Physician involvement in the design and developmental stages of AI platforms should be encouraged to better safeguard patients and their care. We propose best practices to maintain humanism, equity, integrity, and privacy. We present this narrative review to describe previous uses of AI, elucidate its potential for transforming the future of MedEd, and suggest pillars for best practices when using this new technology.

## Student/Trainee Use of AI

### Standardized Exams

Recent studies have indicated that AI chatbots such as ChatGPT demonstrate medical knowledge comparable to a third-year medical student when evaluated by the United States Medical Licensing Exam (USMLE) [13]. With newer versions of ChatGPT being released, the chatbot demonstrated improved performance on USMLE-style multiple-choice questions. Interestingly, it also provided detailed explanations as to why other answer choices were incorrect [14]. AI can also be trained to generate a broad differential diagnosis, highlighting the benefit as an educational tool [13–15]. In addition, generative AI, notably ChatGPT-4, has shown its ability in exams requiring advanced medical knowledge, particularly multiple-choice questions from the European Board of Urology exams [16]. However, in fields such as oncology or, more broadly, areas that require contextualizing information, such as awareness of anatomical position relative to other structures, ChatGPT-4 had notable deficiencies. [16, 17]. Overall, these findings underscore that although AI can assist trainees to better understand learning material or enhance preparation for clinical rotations, we must emphasize that it should not be considered a complete substitute for human cognition and reasoning. AI has been shown to struggle when integrating external and contextual information with sensory and nonverbal cues, and perhaps most importantly, it removes some of the humanistic art of medicine. [2, 5, 13] In addition, other concerns that should be examined include plagiarism, training in AI ethics, and a decrease in critical thinking. [18, 19]

### Surgical Learning

The recent COVID-19 pandemic exposed significant flaws in medical education and resident training. Particularly, an overreliance on in-person instruction with live patients and a lack of sufficient hands-on simulation sessions [20]. In response to the restrictions at the time, many surgical programs adapted their approach to teaching to ensure continued trainee development of surgical skills [20]. AI has facilitated this transition by offering exposure to virtual reality simulations [2, 20, 21]. One of the significant benefits of using this model is that it allows trainees to receive feedback in a low-stakes environment, eliminating potential harm to patients.

For many urologic trainees, using video resources to prepare for surgical cases is a common practice. However, there are concerns that these videos may be unverified and lack peer review; thus, trainees may be studying using inaccurate material [22]. Some programs have been able to use AI to combat the potential misinformation of surgical techniques found in unverified videos while still offering the same level of support and accessibility a trainee would have utilized from an unverified source [23]. At some urology residency programs, AI has been utilized to support residents in reinforcing skills by providing feedback on video recordings of their performance during procedures. These recordings were then integrated into the AI surgical platform, allowing trainees to view annotated video recordings of various urologic procedures and receive personalized performance feedback. This system allowed residents to practice the identification of anatomical landmarks and refine problem-solving skills by allowing trainees to explore alternative procedural methods during independent study.[23, 24]. Additionally, the film room video helps residents save time as it categorizes the type of procedure and annotates various parts of the surgery video for the resident. The AI can help identify various landmarks as well as intraoperative events. Videos that are uploaded to the platform can then be anonymously shared with others to help enhance their learning. The technology also asks for feedback throughout the case, where one can add their own annotations and, at the end of the case, give a “case recap.” These annotations can then be shared with other users to help expand learning even further. This active engagement through AI will likely enhance critical thinking and highlight areas of improvement for both residents and instructors. Additionally, this technology may help improve the overall quality of residency training by facilitating trainees to better prepare for cases and maximizing hands-on learning opportunities with their instructors.

### Personalized Learning

AI has been implemented to personalize the learning experience of students by addressing their unique areas of weakness through automated feedback [2, 19]. AI achieves this by generating multiple-choice questions to reinforce key learning points and provide detailed explanations to aid students'clinical decision-making [14]. When prompted, AI can also assist medical students in creating differential diagnoses that consider multiple domains and pathologies typically beyond their current level of understanding. When learning how to formulate differential diagnoses during preclinical years, students tend to often focus on a singular domain, whereas AI considers more than one domain and presents a broader and more comprehensive differential [13, 14, 21]. This personalized approach will accelerate students’ ability to consider a broader diagnostic paradigm when generating possible differentials. Additionally, using AI to generate differentials could promote learning by teaching students to make connections between certain objective findings and consider pathologies they would not typically have on their own. While AI use in this regard can be beneficial during their training, care must be taken to ensure it does not come at the expense of students developing their own critical thinking skills and ability to make quick clinical decisions for patient care.

### AI as a Search Tool

AI can provide its users with information that is concise and easily accessible, which can be very useful for trainees to identify pathologies based on how a patient presents or even as a point of reference to ask faculty more focused questions. In the clinical setting, trainees may encounter topics or questions they may not be able to answer quickly in pressured situations. Generative AI can provide readily accessible information on desired topic areas and can generate questions to help trainees prepare to field inquiries from faculty about relevant topics [25]. In addition, generative AI tools may be able to present complex concepts in simpler ways for the user to understand, further denoting how useful it is as a quick reference tool.

However, there is a persisting concern that using AI could compromise medical education and research. [26, 27] While generative AI can be helpful, the information quality is not always perfect, as it has been shown to be highly susceptible to misinformation when not closely monitored by users [26]. For instance, in medical writing, AI often fails to answer questions directly and at times lacks detail. An additional concern is that when prompts are not carefully curated, it can lead to inaccurate and unverified information presented in a manner seemingly of higher quality. This emphasizes the responsibility of users to vet information, ensuring its correctness and accuracy. Interestingly, a recent survey at the University of Pennsylvania indicated that ChatGPT received grades of B- to B on an Operations Management exam when used alone, despite ChatGPT’s vast fund of knowledge [28, 29]. This serves as a helpful reminder that, despite the utility of AI, humans nonetheless must verify and validate the information and feedback that AI synthesizes. Collaboration between generative AI and users is likely the best approach to maximize the quality of work produced. In an age of information overload with decreased vetting of information, we must be wary of easy, quick answers that may be inaccurate. Those who are in residency are training to become experts in their field, a skill that AI has still not acquired. An important skill for trainees to develop is exploring and synthesizing vast amounts of information and ultimately generating their own conclusions. Despite the many advantages of AI, it has the potential to compromise MedEd in a variety of ways. This potential has not yet been explored and presents a valuable opportunity to direct future research efforts.

### AI as a Scribe and Research Reading Tool

Medical practice today relies heavily on the electronic medical record (EMR) to document patient encounters. Initially, EMRs were seen as a tool that alleviated the documentation burden; instead, they have contributed to physician burnout, lowered satisfaction, and the current primary care physician shortage [30]. Scribes have been integrated into medical practice, positively impacting workflow, efficiency, and improving doctor-patient interaction [31]. However, not all hospitals can afford scribes. AI has emerged as a promising substitute, displaying scribe-like capabilities to assist physicians in documenting encounters [32]. This gives physicians more time to build rapport, answer questions, or even decompress between seeing patients. Using AI in this way has garnered attention and popularity amongst users. However, there may be potential pitfalls to consider when using AI in this setting. For instance, AI may hallucinate important details of the encounter or even miss out on important nonverbal cues [33]. Strong concerns that an AI scribe could impact learning or lead to dependence are valid. If leveraged appropriately, we foresee AI scribes as not thinking for the trainee but rather alleviating documenting fatigue that inevitably accompanies patient encounters. This will allow increased opportunities to focus on the education piece and rapport-building with patients.

Research is an essential component of MedEd. It is vital that trainees are able to extract meaningful information from research and apply knowledge to various scenarios. AI may facilitate this process by generating research summaries to better identify articles and simplify complex concepts, making knowledge more accessible regardless of background research experience [34]. An important counterpoint to consider is that AI may serve as an intellectual “crutch,” ultimately compromising the learning efforts of trainees. When used appropriately, AI may provide advantages, particularly for those with limited research experience. For instance, using AI to interpret complex, jargon-heavy material often seen in research. In MedEd, AI may be an avenue to facilitate the acquisition of essential research skills when implemented with guidance from senior faculty. This support will better enable trainees to swiftly adopt a clinician’s mindset and develop skills in making meaningful and evidence-based clinical decisions.

## Best Practices

Despite the many benefits of AI use, there are concerns that may limit its adoption in medicine. There is concern for the development of dependency in its users, resulting in a lack of creativity. Further concerns include a decline in critical thinking skills and an increased likelihood of misinformation [10]. This highlights a duality in the outcome of its use, as the benefit may come at a cost to developing the trainee's skillsets and growth. This ultimately brings into focus the best practices of AI use that will safeguard the benefits without compromising ourselves in the process. In this section, we highlight some of the best practices for AI use while denoting concerns for consideration when AI is utilized in this way.

### Research Writing

Research writing is also an area of growing interest due to the prevalence of AI-produced work being submitted for review in academic journals [26, 35, 36]. AI tools such as ChatGPT, as well as platforms such as Grammarly and Turnitin, are particularly adept at detecting AI-produced writing by searching for features that are often definitive, unsupported, and lacking in detail when scrutinized [26]. This and other concerns about the ethical use of generative AI have led to the development of the ChatGPT, Generative Artificial Intelligence, and Natural Large Language Models for Accountable Reporting and Use Guidelines (CANGARU) [36]. This resulted in active efforts to ensure ethical AI use in research and research writing by encouraging authors to indicate AI's role in research or even credit AI as a co-author [37].

This emphasizes the importance of teaching students more appropriate ways to use AI in research and academic writing and will also help address issues of plagiarism. An example of this could be using AI to aid in searching for relevant articles for literature review around a particular topic of interest. AI may also assist students in creating an outline for scientific writing, which will enable them to organize their ideas better and ultimately improve the quality of their work [38]. Despite this, caution is necessary when AI is used to generate scientific papers on its own, as the product may be subject to plagiarism or be riddled with inaccurate supporting evidence [38]. There remain few guidelines, many of which vary between publications, on the extent to which it is valid for AI to write scientific papers. Furthermore, there is no consensus on how AI writing should be identified—whether by granting authorship to AI platforms or adding a disclaimer. This can also be applied to writing within the MedEd context—there are few rules that tend to vary greatly between educational institutions regarding the ability for students to use AI when completing written coursework assignments.

We propose best practices to allow AI integration into research and educational writing while protecting the students'interests and learning. We must recognize the ability of AI to make the writing process more efficient. AI can also create writing that is more accessible or comprehensive. However, MedEd policies must protect the students'ability to learn from their writing, ensure the validity of the final product, and avoid plagiarism. We believe that authors, both in research and as students, should be permitted to use AI to outline content or create first drafts. However, the authors should bear the responsibility to verify the validity of all content, ensuring citations are entered where appropriate and validating that all content is new and without concerns for plagiarism.

### Privacy

AI's reliance on large datasets for learning and executing its desired functions raises concerns for data privacy and protection [35, 39]. This is particularly a concern when we consider AI's integration into healthcare and providing it with access to private patient health information. Even when patient data is anonymized and incomplete, AI may be able to re-identify patients [19]. This ultimately necessitates more robust security measures with the goal of protecting patient information. These can include the implementation of advanced encryption methods and a secure means to protect patient information.

While this is a larger concern for AI, we approach the concern for privacy from the lens of MedEd. When considering AI to track student progress and performance, we propose using third parties to store data, similar to double-blind studies. This eliminates an institution from holding all information. We should be diligent in ensuring that AI is not collecting additional information than what is absolutely required to complete a programmable task. Additionally, this should be executed in a manner that does not violate the Family Educational Rights and Privacy Act (FERPA), which is instituted with the goal of protecting the educational records of students. Though it will not perfectly eliminate the potential of re-identifying students, it can greatly reduce the risk by using AI innovations to maximize educational opportunities for students.

### Misinformation

The presence of misinformation is particularly evident in medical research and academic writing. When prompted, AI can provide content that appears to be of higher quality, but when closely scrutinized for detail, it often reveals inaccuracies and an overall lack of specificity [26]. Studies have indicated that this is likely due to AI's inability to understand inductive reasoning [26, 35].

Despite the many benefits of AI use, these findings emphasize the need for diligence in managing outputs to limit the potential for misinformation. A study assessing ChatGPT's utility in clinical and writing contexts found that ChatGPT could construct a medical note with appropriate formatting, summarize information, and even learn from mistakes when prompted. [40] However, regarding research writing, ChatGPT was found to have a tendency to fabricate evidence that appears to be highly plausible [40]. This phenomenon is described by many as AI hallucination [40]. The presence of hallucinations in AI poses a great risk to the preservation of accurate quality information to users through the generation of misleading findings, further promoting misinformation in MedEd and our society as a whole.

ChatGPT, curating its generated outputs from the entire accessible internet as its fund of knowledge, may make managing misinformation very difficult to control perfectly. Stochasticity is the beauty of generative AI models, yet also a major concern; outputs are not the same between different users or even within a single use with the same prompts. Careful control and monitoring of our input prompts can reduce the risk of misinformation. Further, there is a growing need for a medicine-specific AI tool that can provide the benefits of Generative AI without compromising information quality. This could be accomplished by ensuring that the information that is studied would only come from peer-reviewed resource material.

Conversely, ChatGPT has shown its ability to produce work with quality information when appropriately prompted. Moreover, the information is readily accessible and as understandable as it needs to be for the layperson [41]. Considering this, it is imperative that we engage in continued study as to how we may be able to reproduce this consistently before it is made available to trainees or patients.

### Preservation of Critical Thinking

AI tools were developed as an attempt to replicate human thought, decision-making, and reasoning through learning algorithms [19]. When data and prompts are given, ChatGPT can generate outputs that are reasonable in many contexts. Studies also indicate ChatGPT possesses extensive medical knowledge that can guide patient diagnosis and recommendations for care. However, it struggles to replicate an understanding of important, nuanced factors that guide a physician's reasoning in creating care recommendations at this stage [42]. Some of these factors include personal experiences with similar patients, current events (e.g., a global pandemic), available resources, or even the specific context of the encounter. Physicians are able to synthesize this information to create an individualized plan of care that will meet the unique needs of their patients [42].

This is important as it alludes to the preservation of critical thinking in MedEd as AI continues to be progressively integrated. While AI platforms have value as a quick reference tool, relying too much on generative AI tools could be detrimental to the development of creativity and critical thinking skills [27]. Despite the many advantages to its use, in its current state, AI should not be viewed as a tool to replace human elements but rather as a means of enhancing the efficiency and quality of the work we can produce. Moreover, to safeguard critical thinking in MedEd, we should consider incorporating assessments in training that will challenge students to synthesize information without relying on AI tools.

### Inequity in Education

Disparities in our society typically have far-reaching implications in education and medicine. Regarding education, AI has the capability to personalize the learning experience for students by addressing their individual needs and respective areas of weakness [43]. Not only may this assist students in maximizing their learning potential, but it will be especially beneficial when resources and time with instructors are limited. AI can also enhance accessibility to educational content. Additionally, AI use may aid in reducing the financial burden often incurred by trainees trying to receive quality educational materials. This is achieved by automating the financial aid application process and also by using appropriate algorithms to identify scholarships for students who are most eligible to apply [44].

A worthwhile area to explore is how AI can address educational inequity and accessibility amongst individuals with disabilities. This has become an area of increasing focus amongst developers as efforts are centered on technology that will improve the learning experience of these students. For instance, Microsoft has been working on integrating audio features into ChatGPT to include visually impaired users and complement existing innovations that provide annotations and descriptions to images. [45] Additionally, there are other tools that assist speech interpretation, lip reading, and even detailed image descriptions and annotations for students to utilize as needed.

### Looking Ahead

The potential for AI in MedEd is great despite the concerns for misinformation, as recently, ChatGPT was shown to have substantial capability to create evidence-based responses [46]. AI also presents with vast applications. For instance, surgical residents can learn from enhanced simulation-based learning opportunities that will provide real-time feedback to aid the development of surgical reasoning skills. Many urological residency graduates reported lacking confidence in their training and ability to perform more specialized surgical procedures, including robot-assisted nephrectomies [47]. This should raise serious concerns about how education is delivered to urological trainees and emphasize the urgency in finding ways to better bolster our residents’ confidence through intentional and targeted efforts. AI is uniquely positioned to support these efforts through simulations, VR, and even individualized surgical feedback via an AI platform that curates videos and images for trainees to better refine their surgical skills.

Despite the rapid advancements we have seen in our society, we are still in the early stages of AI integration in medicine. As new AI technologies emerge, we must adapt in a manner that preserves the human element and the quality of our work. For instance, some academic journals ask authors to disclose their use of AI to mitigate plagiarism and uphold standards long synonymous with scholarly writing [48, 49]. The increased popularity of ChatGPT and other AI chatbots among medical students suggests that medical school administrators should find ways to embrace their utility in better preparing students to write for standardized exams and clinical rotations [15]. Another valuable application of AI in medicine has been its utility in educating patients and facilitating follow-up communication in the doctor-patient relationship. It can better answer questions patients may have regarding their health. However, outputs should be monitored due to the tendency for variable results [4]. If greater consistency could be assured with AI use, it could play an important role in providing accurate and reliable results for patients and minimizing misinformation to the wider public.

## Conclusion

AI's adaptable nature has enabled it to be implemented in various ways depending on the individual needs of the user. As AI continues to evolve, its role in medicine and medical education will continue to expand beyond our current expectations. When used ethically, AI can assist in bridging educational disparities through improved accessibility to quality educational material for students, regardless of their location and available resources. Additionally, it can create a safe environment for trainees to hone their skills in diagnostics, decision-making, and even surgery. While these innovations to medicine and medical education are welcomed, it is imperative that these are not void of ethics or independent of human reasoning, but rather focused on augmenting medical study and practice. Moreover, we should ensure the integration of AI into MedEd does not compromise preserving the privacy of the population it was initially intended to assist. As more research is performed to better define AI's role in education, it is vital that more physicians and educators be involved in the design phases to ensure the best practices for AI use are preserved, assuring the continued quality of medical education.

## Key References

- Safranek CW, Sidamon-Eristoff AE, Gilson A, Chartash D (2023) The Role of Large Language Models in Medical Education: Applications and Implications. JMIR Medical Education 9:e50945. Exploring the applications and limitations of large language models such as ChatGPT in medical education from a medical student perspective
- Goldenberg MG (2024) Surgical Artificial Intelligence in Urology: Educational Applications. Urologic Clinics of North America 51:105–115. A review of surgical education and how AI can be used to facilitate learning and the acquisition of surgical skills.
- Alam F, Lim MA, Zulkipli IN (2023) Integrating AI in medical education: embracing ethical usage and critical understanding. Front Med. https://doi.org/10.3389/fmed.2023.1279707. A review of how educators can preserve and promote ethical use of AI as AI continues to garner popularity in medical education
- Ramoni D, Sgura C, Liberale L, Montecucco F, Ioannidis JPA, Carbone F (2024) Artificial intelligence in scientific medical writing: Legitimate and deceptive uses and ethical concerns. European Journal of Internal Medicine 127:31–35. An assessment of the expanding role of AI use in research writing and healthcare.

## Data Availability

No datasets were generated or analysed during the current study.
